# Implementing Suicide Risk Screening in a Virtual Addiction Clinic

**DOI:** 10.1007/s10597-023-01181-3

**Published:** 2023-09-09

**Authors:** Suzette Glasner, August X. Wei, Patrick C. Ryan, Darcy N. Michero, Laura B. Monico, Peyton E. Pielsticker, Lisa M. Horowitz

**Affiliations:** 1 Digital Therapeutics, Inc., 2443 Fillmore Street, San Francisco, CA 94115 USA; 2 Department of Psychiatry and Biobehavioral Sciences, UCLA Integrated Substance Abuse Programs, Los Angeles, CA USA; 3 Office of the Clinical Director, Intramural Research Program, National Institute of Mental Health, Bethesda, MD USA

**Keywords:** Suicide risk screening, Virtual healthcare, Clinical pathway

## Abstract

The purpose of this study was to describe the feasibility of implementing suicide risk screening in a virtual addiction clinic. Suicide risk screening was implemented in a virtual addiction clinic serving individuals with substance use disorders (SUD) using a quality improvement framework. One-hundred percent (252/252) of eligible patients enrolled in the clinic were screened for suicide risk (44% female; M[SD] age = 45.0[11.0] years, range = 21–68 years). Nineteen patients (8%) screened positive for suicide risk. After screening, no patients required emergency suicide interventions (100% non-acute positive). Notably, 74% (14/19) of those who screened positive did so by endorsing at least one past suicide attempt with no recent ideation. Suicide risk screening in virtual addiction clinics yields important clinical information for high-risk SUD populations without overburdening workflow with emergency services. Given the high proportion of non-acute positive screens based on suicide attempt histories with no recent ideation, clinicians may utilize information on suicide attempt history to facilitate further mental healthcare.

## Introduction

Suicide is a growing public health crisis in the United States. Between 2001 and 2020, the suicide rate for all ages in the United States increased by 30% (CDC, 2023). Nearly thirty percent of suicide decedents visited an outpatient mental health provider months, sometimes weeks before their death (Ahmedani et al., 2014), making mental health settings important venues for suicide risk detection. Individuals with a substance use disorder (SUD) are at significantly high risk for suicidal ideation, attempt, and death (Darvishi et al., 2015; Lynch et al., 2020; Poorolajal et al., 2016), with pronounced effects in those with alcohol use disorder (AUD) (Borges et al., 2017) or opioid use disorder (OUD) (Streck et al., 2022). Individuals with an AUD or OUD are 10 and 14 times more likely to die by suicide than the general population, respectively (Rizk et al., 2021). Given these elevated rates, it is critical that practices serving SUD patients can identify and evaluate suicide risk without overburdening practice workflows.

Various settings have implemented suicide risk screening, including outpatient specialty clinics (e.g., diabetes, podiatry) (Aguinaldo et al., 2021; Brahmbhatt et al., 2019; Spector et al., 2022). However, there are currently very few guidelines available to inform best practices for screening and managing suicide risk in virtual settings, which greatly increased in prevalence during the COVID-19 pandemic. Notably, among four million individuals who received SUD treatment, over half utilized virtual healthcare services to maintain access to healthcare amidst pandemic restrictions (SAMHSA, 2021). Recent studies have demonstrated the efficacy of virtual interventions for SUDs (Fiacco et al., 2021; Lin et al., 2019), which lower traditional barriers to treatment and grant various flexibilities (e.g., appointment times, medication management) that enable patient access to their providers.

There are currently no known guidelines for implementing suicide risk screening in virtual addiction clinics. To fill this gap, this quality improvement project (QIP) aimed to determine the feasibility of incorporating a suicide risk screening clinical pathway in a virtual addiction clinic that primarily serves those with AUD or OUD. Feasibility was operationalized in three domains: (1) Positive screen prevalence rate: Are suicidal ideation and behavior common enough in a help-seeking, high-risk SUD population to warrant screening? (i.e., studies of large samples of patients who were universally screened reported positive screen rates ranging from 1.3% to 8.5%) (Horowitz et al., 2010, 2022; Roaten et al., 2021); (2) Acceptability: Do clinic staff and patients find screening acceptable?; (3) Practicality: Can individuals who screen positive for suicide risk be managed effectively without disrupting clinic workflow? A secondary aim was to identify clinical correlates associated with screening positive for suicide risk. Case studies were provided to describe the process and benefits of screening SUD patients for suicide risk in a virtual addiction clinic.

## Methods

### Setting and Participants

This QIP was conducted among a convenience sample of patients ages 18 and older who presented for admission to Pelago, a virtual addiction clinic that provides video-based behavioral counseling, addiction pharmacotherapy, and self-guided psychoeducation and psychotherapy skills training exercises delivered through a smartphone application for individuals with SUD. The program is accessible to individuals in all 50 U.S. states who enroll through their employer. The clinic staff includes licensed drug and alcohol counselors who are certified by the International Certification & Reciprocity Consortium, staff physicians, and nurse practitioners. All patient data, including a multidimensional assessment guided by the American Society of Addiction Medicine’s criteria for placement, care continuity, transfer, or discharge of patients with addiction and co-occurring conditions (e.g., psychotic disorders, acute mania or other primary conditions that warrant immediate and/or higher intensity care), questionnaires, and care documentation were stored in an electronic medical record system. Data used in the present QIP were deidentified and exempt from institutional review board approval.

### Screening Implementation

**Fig. 1:**
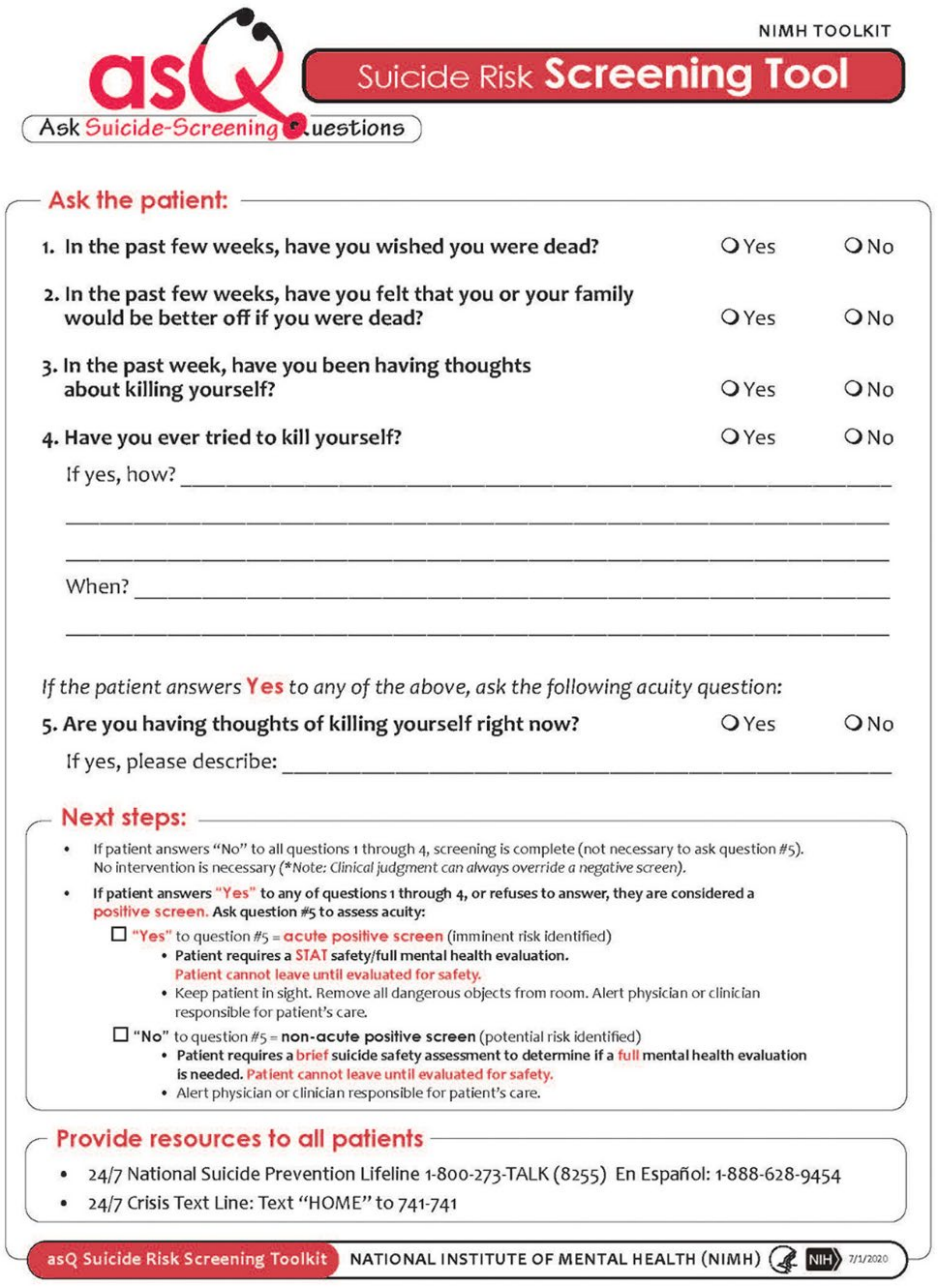
Ask Suicide-Screening Questions (ASQ) tool

In January 2022, Pelago leadership contacted the Ask Suicide-Screening Questions (ASQ) research team at the National Institutes of Mental Health (NIMH) for guidance on adapting and implementing the Telehealth Suicide Risk Pathway (NIMH, 2023) with associated operating procedures. Through an iterative plan-do-study-act (PDSA) approach, Pelago led the QIP over a period of 9 months (January 2022–September 2022). Given the nature of the virtual clinic setting, in which individuals seek treatment services primarily to address substance use, the ASQ screening tool (Horowitz et al., 2012) (Fig. 1) was selected as a brief (20-s), psychometrically sound assessment due to the potential ease of integration into the virtual clinic’s existing diagnostic procedures, with the option for extended evaluation of suicidality among those for whom risk is detected. The ASQ was integrated into the initial multidimensional assessment interview, conducted during the first visit between patients and their assigned licensed drug and alcohol counselor. A Clinical Safety Team channel was established on the Pelago’s instant messaging communication platform (Slack) to designate a weekly safety officer who provided support to counselors if they encountered a patient with active suicidal thoughts. All positive screens were followed up in real-time with the second step in the pathway, the ASQ Brief Suicide Safety Assessment (BSSA).

### Plan-Do-Study-Act Implementation

#### Phase 1: Plan

The initial planning of the QIP involved selection of the ASQ for its validity across various medical settings (Aguinaldo et al., 2021; Horowitz et al., 2012; Horowitz Snyder et al., 2020; Horowitz Wharff et al., 2020) and brief nature, which encouraged ease of administration. The suicide risk screening pathway was modeled after the ASQ Toolkit COVID-19 screening pathway that was adapted for virtual settings (NIMH, 2023).

Counselors and nurse practitioners attended a suicide prevention training webinar in February 2022. The training content included a brief overview of suicide epidemiology, with a focus on individuals with SUD. Best practices according to suicide prevention research and Joint Commission recommendations were presented to facilitate identification of suicide warning signs, risk factors, assessment of the presence/accessibility of lethal means (e.g., firearms, pills) in a patient’s environment, and counseling techniques targeting removal of potentially lethal means. Clinic staff were trained in the administration of the ASQ, interpretation of results, and response to both acute (i.e., current suicidal ideation) and non-acute positive (i.e., no current suicidal ideation) screens.

Clinic staff verbally administered the ASQ as part of the virtual intake evaluation and scored the results in real time. If a patient screened positive, the BSSA was conducted to further triage suicide risk and a safety plan (Stanley & Brown, 2012) was developed and reviewed with the patient. A copy of the safety plan was shared with the patient electronically, with individualized plans for follow-up, care coordination with patients’ other care providers, and mental health referrals when appropriate. To optimize this process, prior to the start of implementation, a list of state-specific crisis and mental health resources was compiled, electronically stored, and shared with all clinic staff.

#### Phase 2: Do

Following initial training, screening was piloted across 4 months (January–April 2022) with all patients ages 18 and older presenting to Pelago. For this phase, clinic staff completed a questionnaire to measure knowledge, attitudes, and experiences related to suicide risk screening. Results were analyzed prior to a second webinar, which involved a refresher training and troubleshooting. Any concerns about the workflow, screening, or assessment process were identified and discussed with the ASQ research team.

#### Phase 3: Study

Following the pilot phase, in July 2022, Pelago clinic staff viewed a refresher training webinar and troubleshooting webinar. This refresher webinar described changes in suicide epidemiology, reviewed qualitative insights from clinic staffs’ feedback surveys, discussed the difference between screening and assessment, and provided updated crisis resources. Any challenges that arose during the pilot phase were discussed in the troubleshooting webinar to ensure staff members felt equipped to continue screening for suicide risk. Some of the questions raised by the staff in the study phase included: (1) Was safety planning necessary with patients who screened non-acute positive because of a prior suicide attempt that was many years in the past, but no current ideation? (2) Which phone-accessible resources were most appropriate for facilitating immediate assessment or intervention for an acute positive screen?

#### Phase 4: Act

After the refresher training, clinic staff screened all patients presenting for admission to the virtual addiction clinic as part of standard of care and held monthly meetings with the ASQ research team to monitor progress. Counselors conducted the BSSA and documented their assessment of risk and corresponding interventions on all positive ASQ screens during the study. Refinements in screening and associated clinical procedures were as follows: (1) In the absence of any current suicidal ideation, safety plans were not implemented for non-acute positive screens whose positivity resulted from a prior suicide attempt that was 5 or more years ago. (2) In addition to the National Suicide Prevention Lifeline and the Crisis Text Line, a list of state-specific crisis intervention resources in each geographical region serviced by the virtual addiction clinic was compiled, stored in a shared drive, and shared with the clinic staff.

### Measures

The ASQ is a four-item suicide risk screening tool validated among pediatric and adult medical patients to detect recent suicidal thoughts and lifetime history of suicide attempts (Horowitz et al., 2012). A positive screen is determined by endorsement of any of the four items, whereas a response of “no” to all four items is considered a negative screen. For any respondents who endorse one of the first four items, a fifth item (“Are you having thoughts of killing yourself right now?”) assesses current suicidal ideation. A positive response to the fifth item indicates an “acute positive” screen, whereas a positive response to any of the first four items with a negative response to the fifth indicates a “non-acute positive” screen.

Demographic information, including age, sex, race, geographical location, and employment status were gathered by the counselor as part of the multidimensional assessment. Clinical information on past trauma history, pain severity, psychiatric medication, and SUD diagnosis were also gathered. Due to the limited sample size and power to detect associations between clinical variables and suicide risk screening outcomes, three clinical variables identified as suicidality risk factors in prior research in populations with substance use disorders were selected for exploratory analysis: trauma history, pain severity, and SUD diagnosis severity.

All staff completed a self-report questionnaire during the pilot phase to examine acceptability and comfortability with suicide risk screening. Items included yes/no questions, short answer response questions, and questions with a Likert scale.

### Data Analysis

Descriptive statistics of patient demographics and suicide risk screening outcomes are reported. Logistic regressions were performed using R Studio 4.2.2 to analyze clinical correlates with suicide risk screening outcome. Qualitative results from clinic staff questionnaires and notes of patients who screened positive are reported.

## Results

### Demographics

Between February 2022 and September 2022, 100% (252/252) of eligible patients enrolled in Pelago agreed to be screened for suicide risk. Race data were not available for 28.2% (71/252) of the sample, as it was not documented in the clinical record for those individuals. The sample was predominantly male (142/252; 56.3%), White (147/181, 81.2%), and ranging in age from 21 to 68, with an average age of 45.0 years (SD = 11.0) (Table 1).

**Table 1:** Demographic and clinical variables of patients presenting to a virtual addiction clinic

Demographics and clinical characteristics	Total sample (N = 252)	ASQ positive (N = 19, 7.5%)
Gender		
Male	142 (56.3%)	7 (36.8%)
Female	110 (43.7%)	12 (63.2%)
Age		
Mean [SD]	45.0 [11.0]	38.1 [8.8]
Range	21–68	26–59
Race		
White	146 (57.8%)	11 (57.8%)
Black	14 (5.6%)	1 (5.4%)
Asian	10 (4.0%)	2 (10.5%)
Hispanic/Latino	8 (3.2%)	–
Missing	74 (29.4%)	5 (26.3%)
Trauma	14 missing	–
Yes	124 (52.1%)	13 (72.2%)
No	110 (46.2%)	5 (27.8%)
PEG (Pain) severity		
Mild	227 (90.1%)	17 (89.5%)
Moderate	21 (8.3%)	1 (5.3%)
Severe	4 (1.6%)	1 (5.3%)
DSM V Dx		
No AUD	10 (4.0%)	1 (5.0%)
Mild	36 (14.3%)	3 (15.0%)
Moderate	55 (21.8%)	1 (5.0%)
Severe	150 (59.9%)	15 (75.0%)

*ASQ* Ask Suicide-Screening Questions, *PEG* Pain, Enjoyment, General Activity, *DSM V Dx* Diagnostic and Statistical, Manual of Mental Disorders (DSM) V Diagnosis

### Screening Outcomes

**Fig. 2:**
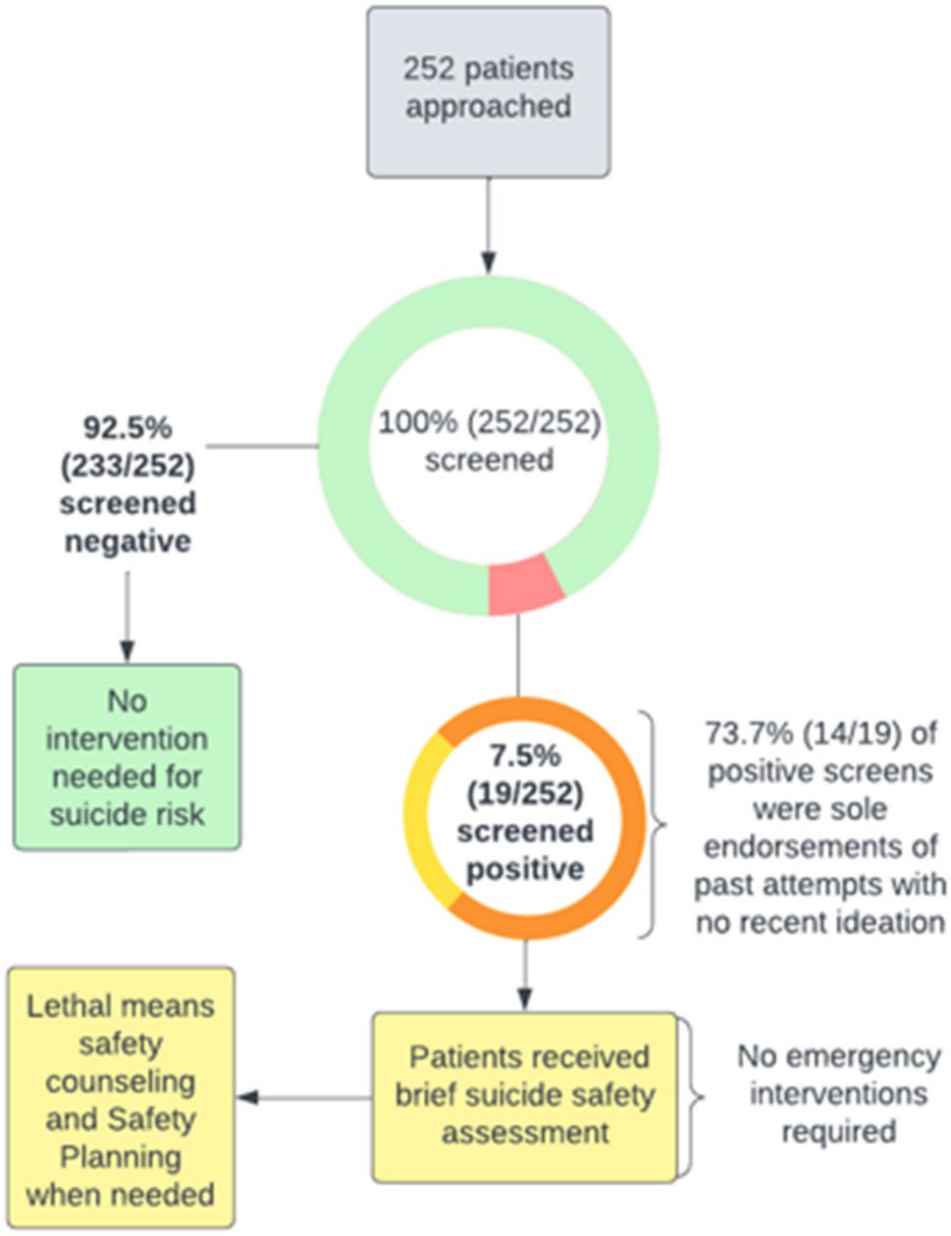
Suicide risk screening workflow. *ASQ* Ask Suicide-Screening Questions

Of the 252 screened patients, 19 (7.5%) answered “yes” to one or more of the ASQ questions, yielding a 7.5% screen-positive rate (Fig. 2). None of the 19 patients who screened positive endorsed the fifth acuity item of the ASQ, indicating that no patients required immediate emergency interventions (100% non-acute positive). 73.7% (14/19) of those who screened positive reported no recent suicidal ideation but endorsed at least one past suicide attempt. Their attempt recency ranged from 2 to 38 years prior to screening, with half (7/14) of attempts being at least 10 years prior.

### Staff Opinions

Six clinicians completed a feedback survey on their experience implementing the ASQ during the pilot phase after the first training. All clinicians (6/6) reported they were comfortable or very comfortable asking patients about past or present suicidal thoughts and behaviors. Four out of six clinicians reported it was acceptable to ask patients about suicidal thoughts when they sought services for substance abuse, citing substance use as a risk factor for suicide. One reported it depended on the circumstances (no details provided) and one did not respond. All clinicians (6/6) were able to list advantages of suicide risk screening in their program, which included knowing a patient’s past history, growing clinicians’ skillsets, and proactively referring patients to mental health services. The majority of the staff (5/6) supported continuing to screen for suicide risk (one clinician was undecided). When asked how screening impacted their overall work environment, three out of six of the clinicians reported no impact on workflow, one was unsure, and two noted screening encouraged discussion among staff on how to respond appropriately to positive screens.

### Clinical Factors

Of those who screened positive, 68.4% (13/19) reported experiencing at least one past traumatic event; 89.5% (17/19) reported experiencing mild pain at the time of their interview; 73.7% (14/19) presented with a severe SUD diagnosis. Twelve patients who screened positive for suicide risk (63.2%; 12/19) were currently taking psychiatric medication.

There were no significant associations between screening positive suicide risk and reports of past trauma (p > 0.3, OR = 1.0[C.I., 0.6–6.0]), pain severity (p > 0.6, OR = 1.4[C.I., 0.3–3.7]), or severity of SUD diagnosis (p > 0.9, OR = 1.0[C.I., 0.6–1.9]) (Table 2).

**Table 2:** Clinical correlate by positive suicide risk screen: logistic regression

Clinical Correlate	Adjusted OR (95% CI)	p-Value
One or more traumatic events	1.03 [0.60–6.03]	0.3
Pain severity	1.35 [0.34–3.74]	0.6
DSM SUD diagnosis severity	1.04 [0.61–1.94]	0.9

Logistic regression model is adjusted for sex and age

### Examples of SUD Patients Who Screened Positive

Table 3 describes examples of clinical features and risk mitigation procedures provided by the for a subset of individuals who screened positive for suicide risk (n = 9) (Table 3). Individuals selected for inclusion in the table were those for whom the most common three themes emerged, either contributing to the screening outcome or facilitating collaborative safety planning with their counselor: (1) psychiatric medication prescription (eight of nine), (2) building rapport between counselor and patient (three of nine), and (3) past suicide attempt (seven of nine). Those who were not included in this subgroup either had a remote suicide attempt more than 10 years prior to the evaluation that did not necessitate current safety planning or had experienced remote but not current or recent suicidal ideation.

**Table 3:** Available descriptions of positive screens and treatment plans

Patient description	Treatment plan
Elderly female patient • Attempted suicide in her 20 s because of an abusive relationship in which she felt trapped • Received outpatient treatment in 2000 for anxiety/depression after first divorce • Prescribed antidepressants but does not actively take them • No suicide plan reported	With knowledge of previous attempt, counselor established a safety plan which included warning signs/symptoms, people/places and resources such as the National Suicide Prevention Lifeline number The patient established three warning signs of a developing crisis: having burdensome thoughts, erratic behavior, and agitated mood. Next, she identified taking a nap as one internal coping strategy that she can use. She identified people and social settings that provide a distraction: her coworker, going to AA meetings, or going to the mall or Hobby Lobby. She identified her coworker as someone she could ask for help and provided his phone number. To make her environment safe and supportive for her mental health, she began with plans to make her home safe, i.e. removing guns and extra supplies of medications. Second, she planned to remove items in her home that trigger her PTSD such as old pictures or items that would remind her of a toxic relationship. Next, she acknowledged that during a crisis she could contact her assigned counselor and/or the Suicide Prevention Lifeline. Finally, she acknowledged that she is important and worth living for
Female patient in mid-20 s • Reported having established PCP • Diagnosed with GAD & MDD • Reports of feeling hopelessness • Family history of SUD • Reports tapering off her anti-anxiety medication	The counselor’s recommendation to engage in the development of a safety plan helped foster trust and rapport with the patient, in addition to the National Suicide Prevention Lifeline number being shared. Patient planned to continue meeting with her wellness coach, whom she was working with outside of the SUD treatment program, in regard to learning how to cope with her anxiety in a healthy manner The patient established four warning signs of a developing crisis: difficulty completing a task, embarrassing herself, feeling unaccomplished, and isolating herself. Next, she identified four coping strategies: grounding exercises, avoiding social media, going outside, and physical exercise. She identified people and social settings that provide a distraction: her brother, her boyfriend, going to the gym and walking around her neighborhood. She identified her brother as someone she could ask for help and included his phone number in her plan. The patient was prompted to create ways to make her environment safe and supportive for her mental health. She stated keeping her home clean to help reduce stress levels. Next, she acknowledged that during a crisis she was willing to contact the Suicide Prevention Lifeline. Finally, she acknowledged that her family is worth living for and important to her
Middle-aged male patient • Denies any current or historic mental health diagnosis • Reported being prescribed anxiety medication but denies taking it • In his ASQ responses, stated he took cocaine and opioids concurrently, resulting in coma • Reports having an established PCP	Patient reported one suicide attempt six years ago. Patient denied a history of thoughts/attempts and was actively engaged in mental health and substance use disorder therapy. A safety plan was not established at this time due to the event occurring more than five years prior to this non-acute positive screen. Patient was deemed stable on Suboxone and was monitored by a counselor and prescribing provider
Middle-aged female patient • Past suicide attempt 20 years ago • At time of assessment, was in counseling and medication treatment for anxiety and depression • Has established routine taking psychiatric medication routinely	Patient established safety plan with counselor despite her attempt being 20 years ago due to having experienced suicidal ideation within two weeks of the ASQ assessment being completed; patient was actively working with her SUD counselor and externally with a provider to treat her mental health; resources including National Suicide Prevention Lifeline number were provided The patient established two prior crisis triggers: traumatic memories and Serzone. She stated the Serzone once made her extremely depressed. Next, she identified three coping strategies: yoga, meditation, and medication. She identified social settings that provide a distraction: getting out of the house to go hiking and walking. She identified her sister, mom, and best friend as people she could ask for help. To make her environment safe and supportive for her mental health, she planned to get out of her house and not spend so much time alone. Next, she acknowledged that during a crisis she could contact her psychiatrist and/or the Suicide Prevention Lifeline
Female patient in early 30 s • Engaged in psychotherapy for anxiety for 10 + years • Diagnosed with Bipolar Disorder 10 years ago before her first suicide attempt • After attempt, her psychiatrist prescribed medication • Other diagnoses in her history include anxiety and depression • Currently taking mood stabilizer and antidepressant medications • Patient reports feeling stable on her psychiatric medications • Denied any suicidal ideation / attempts in the past 10 years	Awareness of present mental health conditions allowed the patient to be open and honest with the counselor about diagnosis and treatment history. Patient opted to complete a safety plan, which included identifying warning signs, coping strategies, personal support, and the National Suicide Prevention Lifeline number The patient established four warning signs of crisis development: sleeping a lot, social isolation, deficient food intake, and feeling down. Next, she identified 5 coping strategies, including letting herself rest and not feeling guilty over it, playing the piano, cross stitching, and playing video games. She identified people and social settings that provide a distraction: her husband and taking a walk around the neighborhood. She identified her husband as someone she can ask for help. To make her environment safe and supportive for her mental health she planned to put alcohol away and out of sight. She acknowledged that during a crisis she could contact her mental health counselor, and/or the Suicide Prevention Lifeline. Finally, she acknowledged that her family, husband, and cat are all worth living for and are important to her
Male patient in mid-30 s • Past history of anxiety, depression, bipolar and PTSD • Reports attending mental health counseling seven years prior • Reported history of prior incarceration and homelessness • Endorsed a history of suicide attempts and suicidal ideation • Family history of SUD	Patient did not share details regarding past suicide attempt and past ideations with counselor. Counselor completed a Safety Plan, which included identifying warning signs, coping strategies, personal support, and the National Suicide Prevention Lifeline number
Male patient in early mid-30 s • Mental health diagnoses of ADHD, bipolar, anxiety, and depression • Reports off and on mental health counseling for a total of four years • Prescribed Gabapentin for anxiety but does not take consistently • History of criminal justice involvement, leading to 18-month court mandated SUD treatment • History of unintentional opioid overdose July 2020 • Reports one prior voluntary hospitalization related to mental health	Patient established a safety plan with the counselor following the initial assessment due to extensive mental health and substance use history. Patient agreed and filled out the plan to the best of his ability. Patient was aware of risk factors and receptive to listing out harm reduction techniques, coping skills, and recognizing the patient's support group to contact in case of an emergency. He was also given the National Suicide Prevention Lifeline number
Male patient in late-20 s • Diagnosed with GAD in 2018 • Taking psychiatric medication daily for anxiety • History of treatment with opioid replacement therapy in 2019–2020 • No PCP established at time of assessment • Reported 3 past suicide attempts in 2017, 2018 and 2019	Patient was cooperative in establishing a safety plan. Patient stated he is working on prioritizing finding a PCP to set up care for future medical needs. Patient had awareness of his present mental health conditions and was open and honest with the counselor about diagnosis and treatment history. The National Suicide Prevention Lifeline was provided at time of safety plan. A follow up safety plan was conducted three months from initial. Patient was stable and doing well with mental health and overall well-being
Female patient in mid-30 s • Diagnosed with Depression and a history of Bulimia • Reports history of 4 inpatient SUD treatment episodes • One prior psychiatric hospitalization in 2011 after a suicide attempt via medication overdose, second suicide attempt in 2022, a few months prior to ASQ administration • Prescribed psychiatric medication for depression • Had a PCP established and reported routine visits	With knowledge of previous and recent attempts, counselor established a safety plan, which included warning signs/symptoms, people/places and resources including the National Suicide Prevention Lifeline number. A follow up safety plan was not completed due to the patient withdrawing from the program

*ASQ* Ask Suicide-Screening Questions, *GAD* generalized anxiety disorder, *MDD* major depressive disorder, *SUD* substance use disorder, *PCP* primary care provider, *PTSD* post-traumatic stress disorder

#### Psychiatric Medication Prescription

Eight patients (eight of nine) reported being prescribed psychiatric medications for depression or anxiety. Five of these patients (five of eight) were actively taking their medication, whereas three had tapered off their medications or denied taking them. Three of these patients (three of eight) reported a history of opioid-related suicide attempts. All these patients (eight of eight) received a Safety Plan.

#### Building Rapport Between Counselor and Patient

Three patients’ (three of nine) notes indicated they were cooperative, open, and honest with their counselor about their psychiatric history. All (three of three) were aware of their present mental health conditions and transparent about their diagnosis and treatment history. One note indicated the counselor’s recommendation to engage in the development of a safety plan helped foster trust and rapport with the patient.

#### Past Suicide Attempt

Seven patients (seven of nine) reported at least one past suicide attempt. Three of these patients (three of seven) had attempted over 10 years ago. Two patients (two of seven) reported multiple past attempts. Safety plans were established for all patients (seven of seven) regardless of how distant in the past their most recent attempt was.

## Discussion

Suicide risk screening was feasibly implemented in a virtual addiction clinic using a QI framework to adapt a Telehealth Suicide Risk Pathway (NIMH, 2023). The 7.5% positive screen rate was high enough to warrant screening, yet low enough as to not overtask resources and staff. All 252 patients approached agreed to complete screening questions, demonstrating acceptability of screening. Clinic staff expressed positive opinions toward screening and supported plans to continue screening SUD patients.

### Relevance to Care

The most important component of suicide risk screening is having a plan in place to respond to patients who screen positive. While some clinicians in the present study expressed concern about providing sufficient support through virtual care for patients expressing acute suicidal thoughts, results demonstrated that most positive screens among this help-seeking SUD population are non-acute and may not require emergency interventions. Furthermore, most of those who screened positive in the present study did so solely on the basis of a past attempt with no current ideation. Existing suicide risk screening clinical pathways address patients who only endorse a suicide attempt history with no passive or active suicidal thoughts. For example, one clinical pathway indicates that if the patient’s attempt was over a year ago, they are seeking mental healthcare, and their suicide attempt history is not a current concern, clinicians are made aware of their suicide attempt history and may exercise judgment to expedite the follow-up brief suicide safety assessment with minimal intervention (Horowitz et al., 2022). Knowledge of suicide attempt histories may affect decision making relevant to the patient’s care (e.g., behavioral treatment intensity, appropriate medication prescriptions, frequency of psychiatric symptom monitoring), coupled with contextual clinical factors that may necessitate more frequent monitoring of suicidal thoughts (e.g., polysubstance use, intravenous drug use). Thus, even when patients who present to virtual clinics are not currently experiencing suicidal ideation, clinicians can utilize information from their mental health history to provide appropriate healthcare.

### Clinical Factors

Consistent with the literature on correlates of suicide risk among individuals with SUD (Pompili et al., 2010), the present investigation revealed a high frequency of trauma exposure, current self-reported pain, a severe SUD diagnosis, and current use of psychiatric medications among those who screened positive for suicide risk. Though these factors did not significantly increase the odds of screening positive for suicide risk, individual risk factors only account for a small proportion of the variance in suicide risk (Oquendo et al., 2006), posing challenges to the identification and management of individuals with suicide risk. Findings highlight the need for more research on how to screen, manage, and monitor patients presenting to a virtual behavioral health clinic setting with combined SUD and psychiatric comorbidities. Moreover, given the well-established impact of alcohol and other drug use on suicide risk, effectively treating the SUD condition is an essential mechanism through which a virtual modality for delivering behavioral health treatment can reduce suicide risk in this population. As such, delivering measurement-based care with frequent SUD symptom monitoring through the combination of both self-report and objective evaluation of substance use (e.g., urine drug screens, breathalyzer assessment) can inform the clinician’s ongoing understanding of current level of suicide risk, enabling ongoing adjustments to the plan of care as indicated.

### Limitations

Several limitations of the present QIP warrant comment. First, this sample draws from a help-seeking, employed subgroup of individuals with SUD, for whom suicide risk is likely lower than that of individuals with a SUD diagnosis who are not seeking treatment or unable to access treatment through their employer (Hom et al., 2015). Given that employment status is associated with suicide risk (Conejero et al., 2016), the positivity rate observed in this study is likely an underestimate of the prevalence of suicidality in SUD treatment centers where greater variation in employment status is observed. Second, the sample had limited race data available, limiting the generalizability of the findings to the broader sample of individuals who were treated in the virtual addiction clinic. Finally, the sample size, constraints conferred by the low frequency event of positivity for suicide risk, and attenuated statistical power limited our ability to detect relationships between clinical variables and suicide screening outcomes.

Nonetheless, this QIP demonstrated the tenability of universal screening for suicide risk without overburdening a virtual addiction clinic, and may serve as a model for implementing a telehealth suicide risk screening pathway in other virtual practices serving high-risk populations.

## Conclusions

Suicide risk screening was successfully implemented in a digital addiction clinic without introducing a burden to clinicians or interfering with workflow. Telemedicine clinicians working with individuals with SUD are well-positioned to ascertain relevant clinical information about suicide attempt history and facilitate further mental health care.
